# The Therapeutic Efficacy of Ankle Mobilization and Advance Physiotherapy in Alleviating Heel Spur and Plantar Fasciitis: A Case Report

**DOI:** 10.7759/cureus.57524

**Published:** 2024-04-03

**Authors:** Manali A Boob, Pratik Phansopkar, Kamya J Somaiya

**Affiliations:** 1 Musculoskeletal Physiotherapy, Ravi Nair Physiotherapy College, Datta Meghe Institute of Higher Education and Research, Wardha, IND

**Keywords:** conventional physiotherapy, proprioceptive neuromuscular facilitation exercise, mulligan mobilization with movement, heel spur, plantar fasciitis

## Abstract

Plantar fasciitis arises from progressive damage of the plantar fascia, which originates at the medial calcaneal tuberosity and associated perifascial tissues. The plantar fascia is made up of three segments that grow from the calcaneus and serve a crucial role in appropriate foot biomechanics. The plantar fascia itself is vital in supporting the arch and absorbing trauma. The heel spur is one of the most prevalent causes of foot discomfort. It is important to determine the most effective technique of therapy based on the emergence of pain at each step of the day. This case report describes the thorough rehabilitation of a 42-year-old mesomorphic female, a yoga instructor, and a recreational runner who presented with heel spur and plantar fasciitis symptoms. In addition to traditional therapy, the patient received advanced physical therapy with an emphasis on Mulligan joint mobilization to lessen discomfort and increase range of motion. The objective was to evaluate the effect of this intervention on several outcome measures, such as the visual analogue scale, balance test, foot functional scale, range of motion, and lower extremity functional scale. Targeted exercises and treatments were incorporated into the comprehensive rehabilitation plan to enhance foot function. The patient received the enhanced physiotherapy intervention well. The outcome measure showed notable gains. This case contributes greatly to our knowledge of the best physiotherapy treatments for those with plantar fasciitis and heel spurs.

## Introduction

Ankle joint and foot-related injury problems are widespread in individuals playing sports, which results in stress fractures, plantar fasciitis, Achilles tendinopathy, ligament injury, and other soft tissue injuries [1]. Plantar fasciitis is a leading cause of foot discomfort that manifests as early morning soreness and plantar region discomfort [1]. Numerous risk factors, such as restricted ankle dorsiflexion, prolonged activity, long-standing obesity, and inadequate foot mechanics, are attributed to plantar fasciitis [2]. By using the windlass mechanism, the plantar fascia is accountable for elevating and sustaining the arch when walking [3]. As a result of the dorsiflexion during terminal stance, the plantar fascia's central band tightens, drawing the forefoot near the heel and raising the arch's height [3]. The most frequent location for heel spurs is the calcaneum, where plantar fascia inserts [4]. The most typical causes of plantar heel discomfort are plantar fasciitis and calcaneal spur. The therapeutic perspective shows there are differences between the two disorders. The most common causes of plantar fasciitis are excess overuse or ligament injury, which results in discomfort and irritation. Injuries to the heel bone result in a calcium deposit that extends beyond the border of the bone and causes heel spurs [5]. The implementation of an ultrasound machine is one of the most traditional approaches. It facilitates the transfer of ultrasonic waves that increase the levels of heat in the tissue and enhance the fascia's extensibility, which stimulates the thermal receptors. An ultrasound's nonthermal functions include controlling membrane characteristics, modifying cell division, and promoting the creation of proteins linked to inflammation and damage healing [6]. There are many different therapeutic technique options, including manual therapy, stretching, strength training exercises, electrotherapy agents, advice about appropriate footwear, and orthotic devices [7]. This case study details a yoga instructor who underwent physical therapy after being diagnosed with plantar fasciitis and a heel spur. This case report describes how joint mobilization and other therapeutic intervention aids in pain relief, enhances ankle joint range, and improves foot mechanics.

## Case presentation

We report a case of a 42-year-old mesomorphic lady who was a yoga trainer and recreational runner. She was referred to the physiotherapist by the orthopedic surgeon as she was suffering from plantar fasciitis. She had complained of persistent and worsening pain in both heels for three months. The pain was sharp and stabbing in nature, and it was further exacerbated on the first initial steps in the morning and after a prolonged period of standing. The pain onset was gradually progressive over the last three months, so it was impacting the patient's regular recreational activities and causing difficulty in maintaining balance while performing activities of daily living. The pain was revealed as the foot off-loaded from the weight-bearing position, and after a few minutes, the pain was completely gone. There was no history of accident, fall, trauma, numbness, tingling, or inflammatory conditions in the lower extremities. There was no relevant family history and no history of any medication or operation for this illness. 

Clinical findings

**Figure 1:**
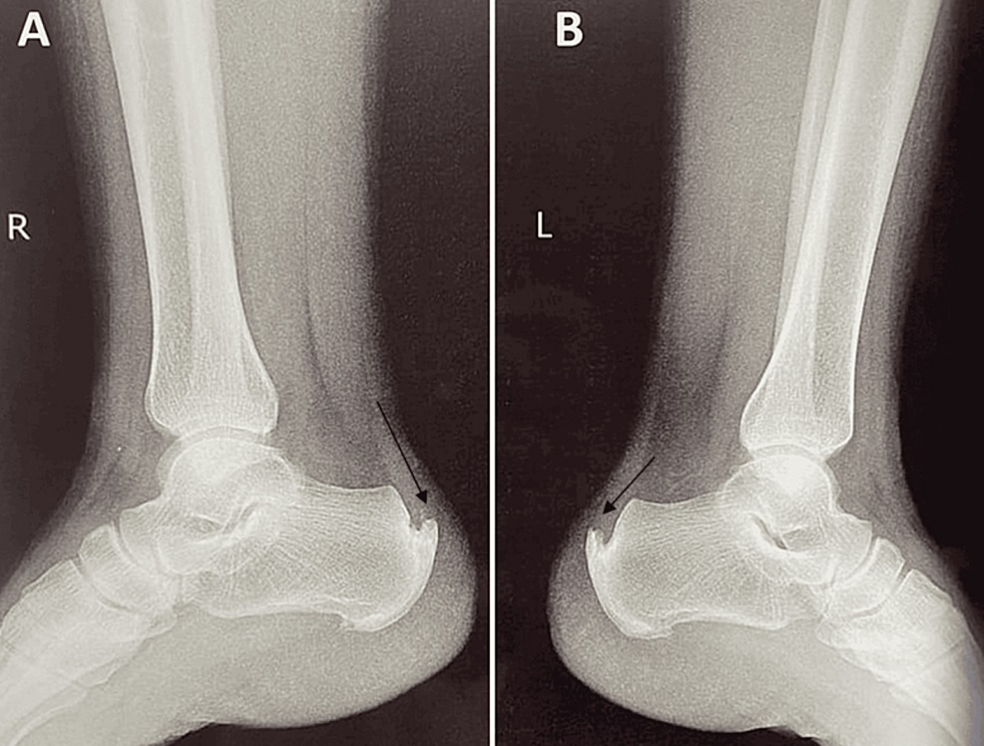
Radiological investigation shows bilateral heel spurs A: X-ray of the right heel; B: X-ray of the left heel

Before the examination, proper oral and written consent was obtained. On inspection, bilateral swelling was observed on the plantar aspect of both the heel. There were no visible redness or signs of skin abnormalities. On palpation, bilateral grade two tenderness was present on the plantar surface of the bilateral heel. There was no palpable mass or nodules. The dorsiflexion range of the bilateral ankles was reduced. The windlass test is positive for the bilateral ankle, which shows severe exacerbating pain on passive dorsiflexion of the great toes. The Tinel's sign was negative as there was no tingling and radiating sensation on the tapping of the posterior tibial nerve. According to the radiological examination, the patient appeared to have bilateral heel spurs on the right (Figure 1A) and left (Figure 1B) sides. The above physical examination and X-ray findings confirmed that the patient was suffering from plantar fasciitis and heel spur. The timeline of this case report is mentioned in Table 1.

**Table 1:** Timeline of the event

Date	Events
02-12-2023	The patient visited the physiotherapy department with complaints of bilateral heel pain
03-12-2023	A radiological investigation was done, which suggested a bilateral heel spur. A physical examination revealed a positive windlass test, which confirmed plantar fasciitis
04-12-2023	The physiotherapy sessions were started
08-01-2024	The end of the physiotherapy rehabilitation and the outcome measures were assessed

Therapeutic intervention

The therapeutic goals of the heel spur and plantar fasciitis intervention were to reduce discomfort, enhance range of motion, treat underlying biomechanical problems, and try to prevent the recurrence of the symptoms. The difficulty of these workouts should be increased gradually and adjusted to the patient's tolerance level. Maintaining consistency is essential to improving range of motion and successfully treating heel spurs and plantar fasciitis. These exercises must be advanced progressively and adjusted to the level of difficulty in accordance with personal tolerance. Table 2 shows a comprehensive, goal-oriented advanced physical therapy rehabilitation program for heel spurs and plantar fasciitis.

**Table 2:** Advanced physical therapy intervention according to the patient's condition

Goals	Therapeutic interventions
Patient education	Educated on the cause of discomfort and the therapeutic action. Patients are being educated about the necessity of proper footwear, particularly during weight-bearing activity. Advising on when and how to change orthotic devices to keep them effective
To relieve pain and minimize the appearance of inflammation	Cryotherapy every ten minutes repeatedly in the day
	Ultrasound is used to enhance tissue healing. The duration is eight minutes with an intensity of 1.8 watts per square centimeter and a frequency of 1 megahertz
	Orthotic insoles were advised to treat foot arch concerns and enhance foot alignment. Orthotics were customized to suit differences in arch height, pronation, and supination
Enhance ankle mobility to improve overall foot function	The Mulligan subtalar mobilization to increase plantarflexion [6]. The patient was sitting in a long sitting position, with the affected leg knee bent. With one hand, the therapist anchored the distal ends of the tibia and fibula and then used the other hand's web space to slide the talus anteriorly (Figure 2A)
	Subtalar joint lateral glide mobilization: The therapist uses one hand to support the distal tibia and fibula, while the patient is lying on the side that is affected and with the leg resting on the pillow. The therapist applies a mobilizing force perpendicular to the ground with one hand while holding the calcaneus, which is distal to the talus (Figure 2B)
	The Mulligan nonweight-bearing mobilization to increase dorsiflexion: The patient was seated in a long sitting position. By holding the belt with both hands and an anchor belt running along the middle of the foot. The patient was undergoing actively aided dorsiflexion with the assistance of a belt. At the same time, the therapist stood in front of them, one hand stabilizing the distal part of the leg and the other providing a posterior glide to the talus with web space (Figure 3)
	The Mulligan weight-bearing mobilization to increase dorsiflexion: The patient's afflicted leg was flat on the splint, and she was in the half-keeled posture. A belt is fastened at the distal end of the leg, wrapped around the therapist's waist, pulled forward, and then provides a posterior glide to the talus with the web space (Figure 4A)
	The Mulligan movement with mobilization with a belt to increase dorsiflexion: The therapist places the patient in a half-kneeling position. A padded belt encircles the patient's distal posterior calf and the therapist's pelvis. The therapist leaned back, adjusted the belt posteriorly, and provided the posterior glide to the talus with web space as the patient performed active dorsiflexion (Figure 4B)
Boost surrounding muscle strength and increase ankle mobility	Isotonic workouts for the plantar and dorsiflexors, intrinsic exercises for the foot, and resistance exercises for the ankle using the theraband advance based on the patient's performance
To improve proprioception and encourage stability while doing weight-bearing exercises	Ankle proprioception neuromuscular facilitation includes reversal of antagonist and dynamic reversal in the form of a diagonal pattern
	Proprioceptive exercises that are both static and dynamic include wobble board standing, single-leg mini squats, and lunges with and without perturbations
Optimal gait mechanics and gait training	Throughout the gait cycle, place special emphasis on a controlled heel strike and toe-off phase. Exercises involving partial weight-bearing and progress to weight-bearing as tolerated. Proper gait mechanics were reinforced by using verbal and visual cues

**Figure 2:**
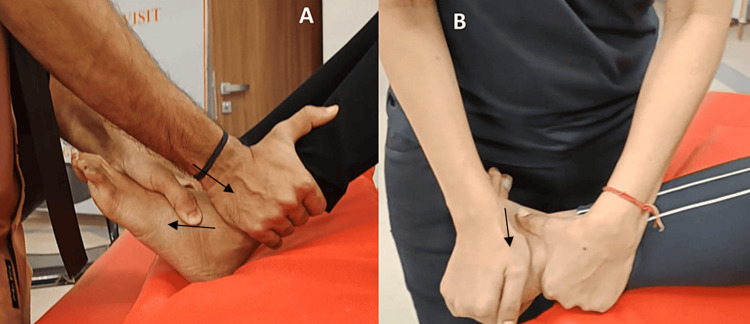
The above images show the mobilization of the subtalar joint A: Anterior glide to the talus to increase the range of plantar flexion; B: subtalar mobilization lateral glide

**Figure 3:**
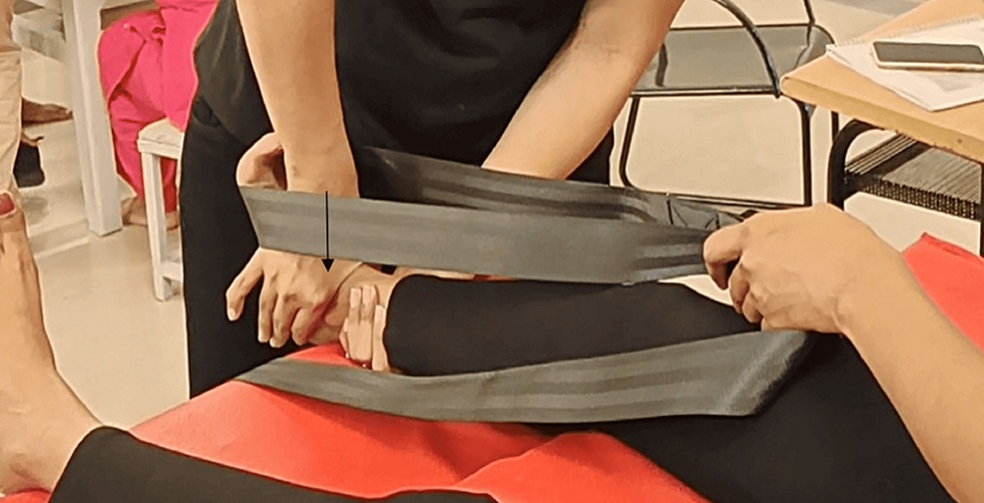
Posterior glide to the talus to increase dorsiflexion in nonweight-bearing position

**Figure 4:**
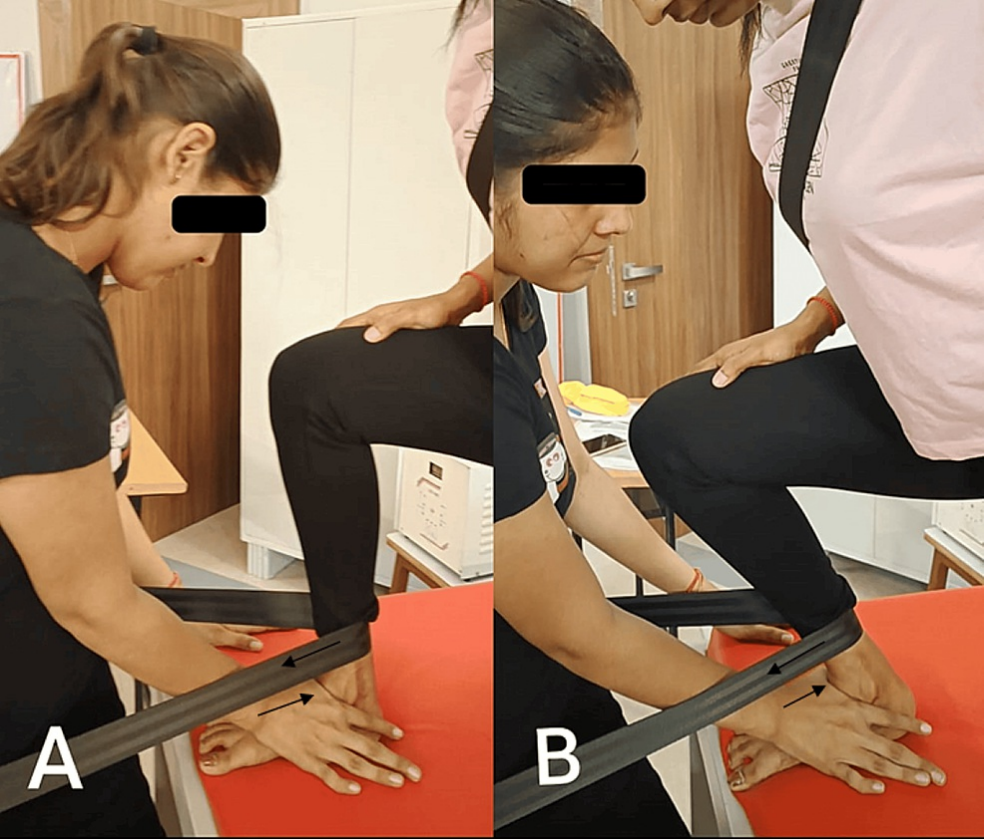
The Mulligan weight-bearing mobilization to increase dorsiflexion A: Posterior glide to the talus in weight-bearing position; B: movement with mobilization to increase dorsiflexion

Outcome measures

Incorporating many outcome measures provided a thorough evaluation of the patient's state, degree of discomfort, functional restrictions, and overall impact on the quality of life of the patient suffering from heel spur and plantar fasciitis. Table 3 shows the outcome measures.

**Table 3:** Pre- and post-intervention outcome measures

Parameters	Pre-physiotherapy (day 1 before the intervention begins)	Post-physiotherapy (after the four weeks of intervention on the last day)
Numerical pain rating scale	Eight out of ten	Four out of ten
Plantarflexion range of motion	30°	50°
Dorsiflexion range of motion	10°	20°
Inversion range of motion	15°	30°
Eversion range of motion	10°	15°
Foot functional index	70%	20%
Y balance scale	54%	80%
Lower extremity functional index	37.8%	76%

## Discussion

Among the most prevalent foot diseases, plantar fasciitis affects around 10% of the normal community at some point in their lifespan. It is common in women, particularly in the 40-60 age range, and in youth sports [7]. The community, general practitioners, sports science specialists, and orthopedists need to be informed about the spectrum of physical therapy practice and the value that physiotherapists provide to the treatment of patients with plantar fasciitis. Referral by medical professionals may be hindered by underappreciation of the advantages of specialist physical therapist care, misconceptions about the genesis and management of plantar fasciitis, or beliefs that the condition is self-limiting with no long-term effects [8]. The arrangement of the domes while walking is largely dependent on the direction of the plantar fascia. Ineffective foot mechanics have an impact on several gait cycle phases. Extended stress irritates the bony tubercle and the plantar fascia. Excessive pronation might decrease the posterior tibialis and lead to the plantar fascia expanding. The imbalance encountered during the propelling phase of ambulation results in lengthening, which inhibits the optimal utilization of the foot's windlass mechanism [9]. Foam rolling is becoming a common technique for self-myofascial release, which is thought to operate on the same therapeutic mechanism as conventional myofascial release. But it necessitates the person applying pressure with their body weight. Both sweeping and direct pressure on the soft tissues are produced by the movement of the foam roller in relation to the tissue structure. By dissolving adhesions, this improves the lubricity of the fascial layer and enhances tissue extensibility [10]. Lee et al. in this instance emphasize how crucial hip muscle strengthening is to the best treatment of individuals with plantar fasciitis who also appear to have a high-arch foot and have periodic pelvic discomfort [2]. Clinicians should attempt to determine hip abductor muscle weakness in patients with plantar fasciitis who seem to have a high-arch deformity. Therapists should also think about doing hip-strengthening activities [2]. Kashif et al. indicated that compared to the traditional physical therapy group that received ultrasound, stretching exercises, and rigid taping, the group of plantar fasciitis patients who received subtalar mobilization with movement, stretching exercises, and rigid taping demonstrated a comparatively stronger improvement in their quality of life and functional disability [6]. The combined benefits of traditional physiotherapy and ankle mobilization indicate a holistic approach that treats the underlying causes of plantar fasciitis and heel spurs as well as their symptoms. This combination approach's effectiveness is attributed to its complex character, which promotes both pain alleviation and functional improvements that are essential for the patient's overall health.

## Conclusions

In this case report, advanced physical therapy intervention, more precisely, the Mulligan joint mobilization, was used to successfully treat plantar fasciitis and heel spurs in a yoga trainer. The results show that this all-encompassing rehabilitation method is effective since it reduces pain and improves range of motion, foot function, and dynamic balance. This instance adds significantly to our understanding of successful physiotherapy treatments for those with plantar fasciitis and heel spurs, especially those who participate in physically demanding hobbies like running and yoga.
